# *Abrus precatorius* Leaves: Antioxidant Activity in Food and Biological Systems, pH, and Temperature Stability

**DOI:** 10.1155/2014/748549

**Published:** 2014-01-30

**Authors:** Vanitha Reddy Palvai, Sowmya Mahalingu, Asna Urooj

**Affiliations:** Department of Studies in Food Science and Nutrition, University of Mysore, Manasagangotri, Mysore 570006, India

## Abstract

Natural antioxidants present in foods and other biological materials have attracted considerable interest because of their presumed safety and potential nutritional and therapeutic effects. Antioxidant constituents of plant materials act as radical scavengers and convert the radicals to less reactive species. *Abrus precatorius* (AP) was analyzed for its proximate and phytochemical composition. The leaves were extracted with methanol (ME) and analyzed for antioxidant activity by radical scavenging method, reducing power, ferric reducing capacity, and *in vitro* inhibition of Fenton's reagent-induced oxidation in oil emulsion and microsomes. In addition, the effect of temperature (100°C, 15, and 30 min) and pH (4.5, 7, and 9) C on the antioxidant activity of ME was investigated. The leaves were rich in total polyphenols, flavonoids, *β*-carotene, glutathione, *α*-tocopherol, and ascorbic acid. The ME exhibited varying degree of antioxidant activity in a dose-dependent manner. The AP exhibited more inhibition of oxidation in microsomes (73%) than compared to oil emulsion (21%). Heat treatment resulted in an increase of radical scavenging activity of extract (28% to 43%). At pH 4.5 the extract exhibited more antioxidant activity and stability compared to pH 7 and 9. Data indicates that potential exists for the utilization of *Abrus precatorius* as a natural antioxidant.

## 1. Introduction

Natural antioxidants present in food and other biological materials have attracted considerable interest because of their presumed safety and potential nutritional and therapeutic effects. Because extensive and expensive testing of food additives is required to meet safety standards, synthetic antioxidants have generally been eliminated from many food applications. The increasing interest in the search for natural replacements for synthetic antioxidants has led to the antioxidant evaluation of a number of plant sources [1] especially spices and herbs [2]. A large number of plants have been screened as viable sources of natural antioxidants including tocopherol, vitamin C, carotenoids, and phenolic compounds which are responsible for maintenance of health and protection from coronary heart diseases and cancer [3, 4].

In present time, medicinal plants as rich source of natural bioactive components are given priority to study their antioxidant activity and explore their utilization in treatment of diabetes mellitus, dyslipidemia, and cardiovascular diseases. Our team had explored some medicinal plants, namely, *Aegle marmelos* [5], *Morus indica* [6], *Moringa oleifera* [7], *Raphanus sativus* [8], and *Psidium guajava* [9] for their antioxidant activity and stability. Before exploring a medicinal plant, there is a need to analyze the plant for its phytochemical composition, antioxidant activity, and its stability.

In the present experiment medicinal plant, namely, *Abrus precatorius* (common name: Rosary pea), was selected to study the proximate, phytochemical composition, antioxidant potency, and stability in its methanol extract.

## 2. Materials and Methods

### 2.1. Plant Material

The selected plant material *Abrus precatorius (AP)* leaves were collected from Western Ghats of Karnataka. The samples were identified by Dr. Janardhan, Department of Studies in Botany, University of Mysore, and voucher specimen was retained in the laboratory for future reference. The leaves were washed, dried in the oven overnight at 50°C, powdered, passed through 60 mesh, and stored at 4°C till further use.

### 2.2. Proximate Composition

In the dry powder moisture content was determined by using moisture analyser (Metler Toledo MJ33, Lab systems Bangalore, India). Fat, protein, ash, total fiber (soluble and insoluble fiber), iron, calcium, and phosphorus were estimated as per the AOAC [10].

### 2.3. Determination of Phytochemical Components

In the dry sample, different antioxidant components were estimated using standard methods. Ascorbic acid was determined according to the titrimetric method using 2.6-dichlorophenol-indophenol dye [11]. *α*-Tocopherol was extracted by direct saponification of dried sample and estimated based on formation of a red complex from reaction of *α*,*α*′-bipyridyl with ferrous ion due to reduction of ferric ion by tocopherol [12]. *β*-Carotene was quantified by open column chromatography, followed by measuring the absorbance of elute at 450 nm against standard *β*-carotene [11]. Reduced glutathione was determined based on the development of a yellow compound due to reaction of DTNB (5,5′-dithiobis-2-nitrobenzoic acid) with compounds containing sulfhydryl groups [13]. Total phenols were extracted from a weighed portion (50–500 mg) of dried sample with 5 mL of 1.2 M HCl in 50% aqueous methanol for 2 h and with 70% acetone shaken for 2 h and analyzed by Folin-Ciocalteau micromethod [14]. Results were expressed as mg or g of gallic acid equivalent. The flavonoid content was determined by pharmacopoeia method using rutin as a reference compound. The amount of flavonoids in plant extracts in rutin equivalents (RE) was calculated by the following formula:

(1) $$\begin{array}{c} X=\frac{A\cdot m_{o}\cdot 10}{\left(A_{o}\cdot m_{o}\right)}, \end{array}$$

where *X*-flavonoid content, mg/g; *A*—the absorption of plant extract, *A*_*o*_—absorption of standard Rutin solution; *m*—weight of plant extract (g); *m*_*o*_—weight of the Rutin in the solution (g) [15].

### 2.4. Extraction of Antioxidants

A 15 g sample was extracted with 50 mL methanol for 6 h in a mechanical shaker. The extracts were filtered and filtrates were evaporated at 40°C under reduced pressure to dryness in a rotary evaporator (Superfit, India). The residue of each extract was stored in airtight container at 4°C until used.

### 2.5. Radical Scavenging Activity (RSA)

The ability of extracts to scavenge 2,2-diphenyl-1-picrylhydrazyl (DPPH) radicals was determined according to the method of Blois, 1958 [16].

### 2.6. Reducing Power Assay (RPA)

The ability of extracts to reduce iron (III) to iron (II) was determined as per Yildirim et al., 2003 [17].

### 2.7. Ferric Reducing Antioxidant Power (FRAP)

Measurement of ferric reducing antioxidant power of the herbal extract was carried out based on Benzie and Strain procedure, 1999, [18].

### 2.8. Inhibition of Lipid Oxidation

The antioxidant activity of the above extracts (300–500 *μ*g) was determined in an edible oil emulsion and liver microsomes by modified method of thiobarbituric acid-reactive-substances TBARS. This method determines the resistance of lipid or lipid emulsions to oxidation in the presence of the antioxidant. The malondialdehyde (MDA) or TBARS assay has been used extensively since the 1950s to estimate the peroxidation of lipids in membrane and biological systems [19].

#### 2.8.1. In Oil Emulsion

Five grams of oil (sunflower) was weighed and an emulsion was prepared in phosphate buffer of 0.01 M, 7pH, and Tween 20. To different concentrations (300–500 *μ*g) of extracts, 300 *μ*L of emulsion and 450 *μ*L of Fenton's reagent were added and volume was made up to 2 mL and incubated at 50°C for 6 hrs. A control was run without extract. After incubation, 1 mL of thiobarbituric acid (TBA) was added and heated in boiling water bath for 30 min and cooled immediately. The inhibition of lipid peroxidation in sun flower oil was determined by TBA, in which the secondary oxidation products (TBARS, expressed as MDA equivalents) formed by oxidation of oil were determined by measuring the absorbance at 532 nm [20].

#### 2.8.2. In Microsomes

A healthy male adult rat was obtained from the Central Animal House, Department of Zoology, University of Mysore, after availing clearance from the University Animal Ethics Committee of University of Mysore (no. MGZ/2620/2011-12; dated: 31.01.2012). The rat was sacrificed and liver was immediately removed from the rat and placed in cold triethanolamine HCl buffer (0–4°C) at pH 7.4. The liver was thoroughly chilled and homogenized. The homogenate was centrifuged in ultra-centrifuge at 60,000 g for 60 min. The 60,000 g microsomal pellet was then rinsed with buffer and frozen in a freezer (−20°C). The suspended microsomes to be used for the assay were diluted with buffer to give a protein concentration of 5–10 mg/mL [21].

To an aliquot of liver microsomes (1 mg protein concentration), Fenton's reagent and methanol extract (at varying concentration 300–500 *μ*g) were added and incubated at 50°C for 2 hrs. After incubation, 1 mL of TCA (10%) and 1 mL of TBA were added and heated in boiling water bath (15 min) and cooled in ice bath immediately. After cooling, 2 mL of butanol was added and the pink color developed was read at 535 nm. A control was run without plant extract [22]

(2) Antioxidant  activity%=Absorbance  of  control−Absorbance  of  sampleAbsorbance  of  control×100.

### 2.9. Heat and pH Stability

The extracts were heated in a boiling water bath for 15 to 30 min and the residual antioxidant activity was determined by RSA, using DPPH, as described previously. For pH stability, the extracts were incubated for 72 h at pH 4.5, 7.0, and 9.0 and the residual antioxidant activity was determined during the incubation period at different time intervals (0 min, 24, 48, and 72 h) using RSA method [23].

## 3. Statistical Analysis

Mean values of triplicates (*n* = 3) were subjected to one-way ANOVA and Tukey's multiple comparison tests using SPSS software (version 11) (*P* < 0.05).

## 4. Results and Discussion

### 4.1. Proximate and Phytochemical Composition

*Abrus precatorius* (AP) was found to be good source of nutrients and phytochemicals such as *β*-carotene, glutathione, *α*-tocopherol, ascorbic acid, and phytochemicals, namely, total polyphenols and flavonoids (Table 1).

### 4.2. Radical Scavenging Activity

**Figure 1:**
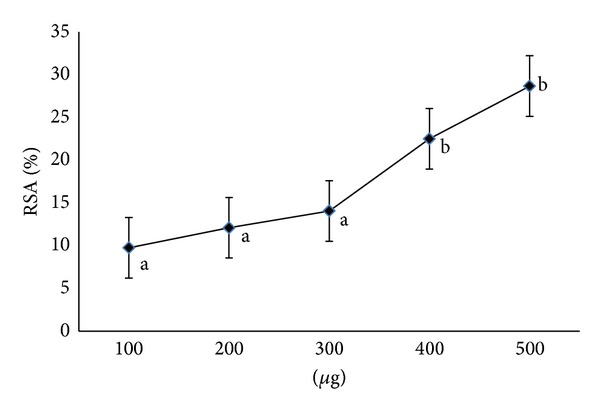
Radical scavenging activity of *Abrus precatorius* methanol extract. Values are expressed as mean of triplicates (*n* = 3) (*P* ≤ 0.05).

The % radical scavenging activity of AP was measured at 100–500 *μ*g (Figure 1). From the figure, it is evident that the scavenging activity of samples was dose dependent, that is, 100–500 *μ*g.

### 4.3. Reducing Power Assay

**Figure 2:**
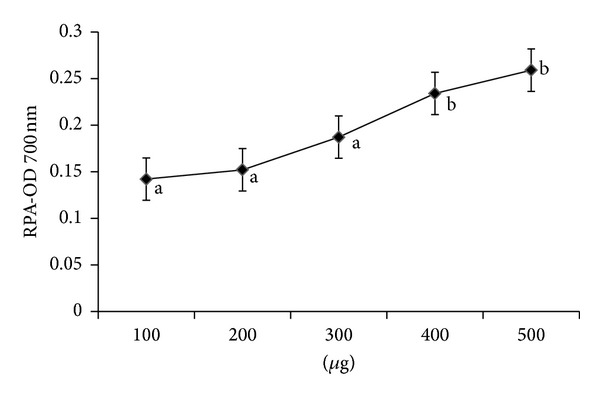
Reducing power assay of *Abrus precatorius* methanol extract. Values are expressed as mean of triplicates (*n* = 3) (*P* ≤ 0.05).

The reducing power of a compound is related to its electron transfer ability and may therefore serve as a significant indicator of its potential antioxidant activity. The electron donating capacity was measured at 100–500 *μ*g (Figure 2). The reducing power of the samples was dose dependent which had ranged between. In the present study, the ability of extract to reduce iron (III) to iron (II) was compared with that of a standard (ascorbic acid) and the reducing capacity was inferior to that of ascorbic acid.

### 4.4. Ferric Reducing Antioxidant Power (FRAP)

**Figure 3:**
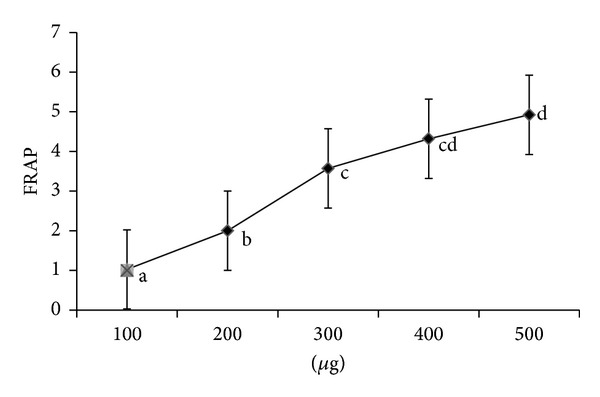
Ferric reducing antioxidant power (FRAP) of *Abrus precatorius* methanol extract. Values are expressed as mean of triplicates (*n* = 3) (*P* ≤ 0.05).

The change in absorbance at 593 nm owing to the formation of a blue colored Fe (II)-tripyridyltriazine compound from colorless oxidized Fe (III) is formed by the action of electron donating antioxidants. This represents an electron exchange reaction. Sample showed high reducing power at lower concentration (100 *μ*g) and increased with the concentration (Figure 3).

### 4.5. Inhibition of Lipid Peroxidation

**Figure 4:**
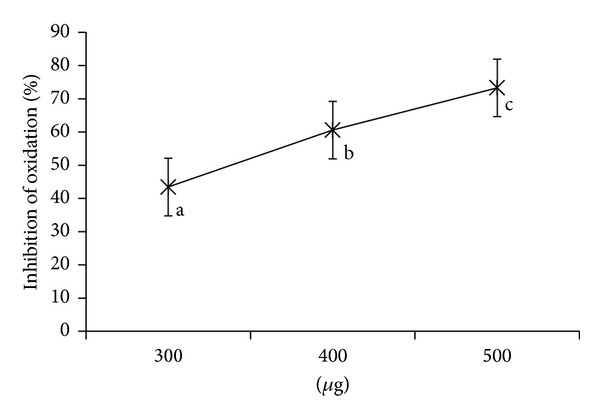
Inhibition of oxidation in edible oil emulsion by *Abrus precatorius* methanol extract. Values are expressed as mean of triplicates (*P* ≤ 0.05).

**Figure 5:**
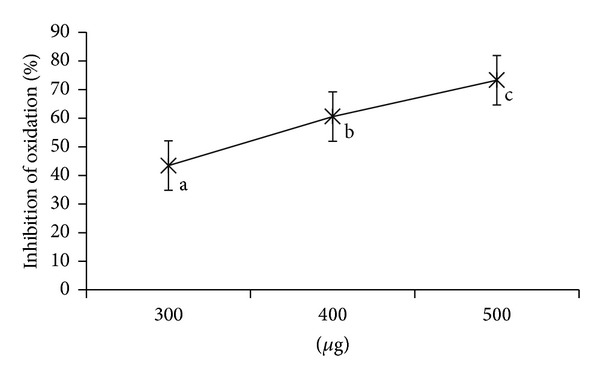
% Inhibition of oxidation in microsomes by *Abrus precatorius* methanol extract. Values are expressed as mean of triplicates (*P* ≤ 0.05).

Lipid oxidation is a process in which PUFA undergoes oxidative damage resulting in the formation of lipid-derived radicals such as alkoxy and peroxyl radicals further causing membrane damage and cellular injury. In biological systems, antioxidants are capable of stabilizing or deactivating free radicals before they attack cells [24]. The sample exhibited high activity in case of microsomes than the oil emulsion (Figures 4 and 5). The rate of inhibition of the oxidation by AP in microsomes was 300 *μ*g, 43.43 ± 9; 400 *μ*g, 60.58 ± 4.79; 500 *μ*g, 73.29 ± 6.84.

Plant-derived antioxidants have been shown to function as singlet and triplet oxygen quenchers, free radical scavengers, peroxide decomposers, enzyme inhibitors, and synergists. The most current research on antioxidant action focuses on phenolic compounds such as flavonoids. Fruits and vegetables contain different antioxidant compounds, such as vitamin C, vitamin E, and carotenoids, whose activities have been well established [25].

Glutathione, tocopherol, *β* carotenoids, flavonoids, and so forth come under second line defense antioxidants. *β*-carotene is an excellent scavenger of singlet oxygen. Vitamin C directly interacts with radicals like O_2_^−^ and OH^•^. Glutathione is a good scavenger of many free radicals like O_2_^−^ and OH^•^ and various lipid hydroperoxides and may help to detoxify many inhaled oxidizing pollutants like ozone, NO_2_ and cigarette smoke in respiratory tract. Vitamin E scavenges peroxyl radicals intermediates in lipid peroxidation and is responsible for protecting PUFA present in cell membrane and LDL against lipid peroxidation [26]. On the whole these phytochemicals and antioxidants bestow high medicinal activities of AP.

Results presented here clearly indicate that the methanol extract of AP with some percentage of polyphenols exhibited antioxidant activity in different *in vitro* models. In the process of oxidation, free radicals are considered to play a cardinal role in numerous chronic pathologies. The RSA of AP is comparatively less than the antioxidant activity of few other medicinal plants such as *Aegle marmelos* [7], *Moringa oleifera* [5], *Morus indica* [27] and in case of* Moringa oleifera* correlation between the polyphenol content and RSA was reported [5]. However, the polyphenol content of AP methanol extract is less than the polyphenol content of above medicinal plants.

Binding of metal ions, such as iron, *in vivo* is an antioxidant action of itself, preventing metal ion catalyzed generation of reactive species [24]. In reducing assay, the yellow color of the test solution changes to various shades of green and blue, depending on the reducing power of each compound. The presence of reducers (i.e., antioxidants) causes the reduction of the Fe^3+^/ferricyanide complex to the ferrous form. Therefore, measuring the formation of Perl's Prussian blue at 700 nm can monitor the Fe^2+^ concentration. The ability of AP methanol extract to reduce iron (III) to iron (II) is inferior than the standard (ascorbic acid—0.973), *Aegle marmelos* [7], and *Morus indica* [6] and superior to *Raphanus sativus* [8]. The reducing power of a sample is due to their hydrogen-donating ability. Accordingly, AP might contain higher amounts of reductone, which could react with free radicals to stabilize and block radical chain reactions.

Processed foods containing fats and oils oxidize slowly during storage; various oxidation products cause rancidity and deterioration of the sensory and nutritional property of the food products. Auto-oxidation of fats and oil in processed foods may be prevented by use of oxidation inhibitors or antioxidants. To protect the cells and organ systems of the body against reactive oxygen species, humans have evolved a highly sophisticated and complex antioxidant protection system that functions interactively and synergistically to neutralize free radicals. Thus, antioxidants are capable of stabilizing or deactivating free radicals before they attack cell [28]. Vitamin E scavenges peroxyl radicals intermediates in lipid peroxidation and is responsible for protecting PUFA present in cell membrane and LDL against lipid peroxidation. The polarity of phytochemicals plays a key role in exhibiting antioxidant role at lipid phase especially at unsaturation site, which influences the chain breaking reaction [24]. Presently, in methanol extract of AP antioxidants with high partition coefficient may be distributed hydrophobic compartments for the protection of lipids. AP was more effective in inhibiting the oxidation in microsomes compared to oil emulsion. Similarly, results were found in case of *Morus indica* [6]. Here, the use of two lipid systems was helpful in studying the inhibition potency of different extracts in food and biological level.

### 4.6. Stability of AP Extracts to High Temperature (100°C; 15 and 30 min)

**Figure 6:**
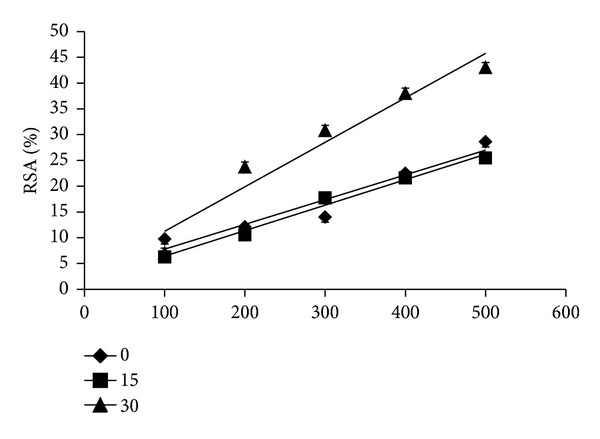
Stability of *Abrus precatorius* extract to high temperature (100°C; 15 and 30 min). Values are expressed as mean of triplicates.

It is well known that many factors such as antioxidant concentration, temperature, and pH of the media processing treatments influence the antioxidant activity. Radical scavenging activity of the heat treated sample extract is shown in Figure 6. After 15 min of heat treatment, a slight decline in the activity was observed at all the concentrations except at 300 *μ*g which showed an increase in the activity. A significant increase was seen at the end of 30 min at concentrations greater than 100 *μ*g. This may be due to the activation of antioxidant components after 30 min of heat treatment. In another study, extracts of drumstick leaves, mint leaves, and carrot were subjected to heat treatment at 100°C (15 min), which resulted in a significant decrease in antioxidant activity (AOA) in drumstick leaves (DL) extract while no difference was observed in AOA of carrot extract. The significant increase in AOA due to thermal processing occurred in mint leaves extract. Thermal processing can induce the formation of novel compounds with antioxidant properties or improve the AOA of naturally occurring antioxidants [29]. Another study reports that WE (water), ME (methanol), and EE (ethanol) extracts of *Aegle marmelos L* treated at 100°C for 15 min showed varied stability; WE showed maximum stability as measured by RSA compared to ME and EE after heat treatment [7]. Similar results were found in case of different extracts of *Raphanus sativus* [8] and *Moringa oleifera* [5].

### 4.7. Stability of AP Extract to Different pH

**Figure 7:**
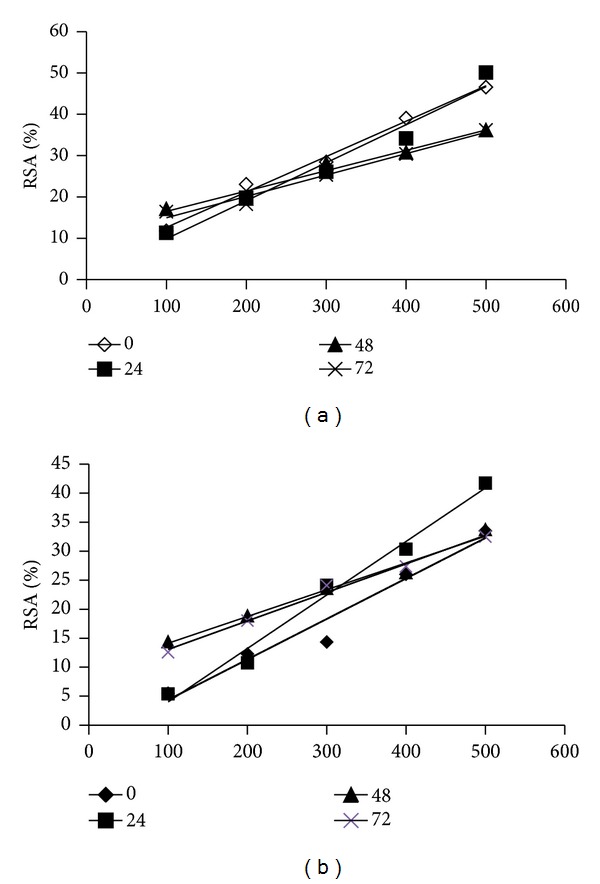
(a) Stability of *Abrus precatorius* extract to pH 4.5, values are expressed as mean of triplicates. (b) Stability of *Abrus precatorius* extract at pH 7, values are expressed as mean of triplicates.

The antioxidant stability of sample extract to different pH (4.5, 7.0, and 9.0) was analysed by radical scavenging activity (Figure 7(a)—pH 4.5 and Figure 7(b)—pH 7.0). At pH 4.5 and 7.0, the extract at different concentrations and time intervals has shown varying antioxidant activity. The influence of pH 4.5 on the stability of AP methanol extract is shown in Figure 7(a), and at the end of 72 h, a decrease in radical scavenging activity was observed. However, the decline in the activity was not linear. The maximum antioxidant activity observed was 50% at 500 *μ*g.

At pH 7.0, after 72 h a significant increase in RSA was observed up to 300 *μ*g and subsequently the RSA decreased at higher concentrations (400 and 500 *μ*g). However, the decline in RSA was not significant compared to the zero min. At pH 9, the extract did not exhibit RSA and the absorbance was more than that of control; this may be due to the structural denaturation of natural antioxidant components and similar results were found in fenugreek seeds and ginger rhizome extracts [30]. In *Aegle marmelos* [7] and *Rapahnus sativus* [8], the antioxidant activity decreased by increasing the pH of media. The antioxidant activity of different extracts from cocoa byproducts was found to be higher at alkaline pH [31]. In an earlier study, it was observed that extracts from mint and carrot leaves showed higher antioxidant activity at pH 9 than pH 4 [32]. These differences might be due to different samples used, various compounds being extracted in each case, and the method used to evaluate antioxidant potency.

## 5. Conclusion

Results of the present study reveal that the *Abrus precatorius* is a potential source of antioxidants which are responsible for the antioxidant activity. The stability to heat and pH of the different extracts of AP leaves extracts with strong antioxidant activity indicates its scope for utilization in food and biological systems. In addition to being consumed as healthy antioxidants, the compounds present in AP that are responsible for antioxidant activity could be isolated and then used as food additives to delay the oxidative deterioration of foods and as nutraceutical in food formulations against degenerative diseases.

## Figures and Tables

**Table 1:** Proximate and phytochemical composition of the *Abrus precatorius*.

Parameter	g/100 g dry basis	Phytochemicals	g/100 g dry basis
Moisture	10.485 ± 0.049	Polyphenols	1.06 ± 0.06
Protein	5.875 ± 0.08	Flavonoids∗	1.325 ± 0.085
Fat	4.40 ± 0.14	Glutathione∗∗	375 ± 176.77
Soluble fiber	6 ± 0.09	*α*-Tocopherol∗	36.6 ± 3.05
Insoluble fiber	2 ± 0.15	Tannins∗	1160 ± 0.076
Ash	8.968 ± 0.97	*β*-Carotene∗∗∗	1260.0 ± 0.05
Calcium∗	1039.5 ± 5	Alkaloids∗	1100.0 ± 0.03
Phosphorous∗	183.20 ± 10.31	Total saponins	0.244 ± 0.01
Iron∗	54.58 ± 0.58	Steroidal saponins	0.186 ± 0.028
		Vitamin C (fresh basis)	0.443 ± 0.011
		Vitamin C (dry basis)	0.643 ± 0.04

*mg/100 g, ∗∗mmol/100 g, and ∗∗∗*µ*g/100 g dry basis.

Values are mean of triplicates (*n* = 3).
